# The Background of a Patient Undergoing Long-Term Periodontal Disease Maintenance Using Step for Coding and Theorization: A Case Report

**DOI:** 10.1155/2024/7941392

**Published:** 2024-10-03

**Authors:** Kosuke Muraoka, Masaki Morishita, Taiji Nakamura, Keisuke Nakashima, Shuji Awano

**Affiliations:** ^1^ Sciences of Oral Functions Department Division of Clinical Education Development and Research Faculty of Dentistry Kyushu Dental University, Kitakyushu, Japan; ^2^ Science of Oral Functions Department Division of Periodontology Kyushu Dental University, Kitakyushu, Japan

**Keywords:** consultation behavior, dentistry, maintenance, periodontal disease, periodontal treatment, periodontology, qualitative research

## Abstract

Periodontal maintenance is crucial for long-term periodontal stability, but some patients do not undertake maintenance following their initial treatment. To date, the motivations, backgrounds, and concerns of patients who underwent maintenance have not been researched. Therefore, we analyzed the subject's intentions and the behavior of the patient affected by periodontal maintenance using Step for Coding and Theorization (SCAT). The subject was a 50-year-old woman diagnosed with periodontitis. Periodontal therapy included oral hygiene instruction, scaling, root planing, and periodontal surgery. She has been continuing maintenance for 21 years. An interview was conducted on a one-to-one basis, between the patient and the surgeon, using a semistructured interview. The contents of the interview included her reasons for visiting the university hospital and her reasons for continuing maintenance. For the qualitative analysis using SCAT, the vocabulary obtained from the interview was filled out in a SCAT form. We determined that the relationship between the dental personnel and the patient was good, and we could convey the importance of maintenance to the patient. Appropriate periodontal treatment and the maintenance of a good relationship with the dental health oral care provider are key factors in improving the rate of maintenance visits and ensuring long-term periodontal stability.

## 1. Introduction

Maintenance after periodontal treatment is very important. Patients on periodontal maintenance have more tooth loss as the periodontal condition increases. If the recall period for periodontal maintenance is long-term, tooth loss increases [1]. Therefore, periodontal maintenance noted the effectiveness. However, some patients do not wish to undergo maintenance.

Periodontal treatment is different from other forms of dental treatment and requires periods of communication with the patient focusing on issues such as plaque control instruction. Therefore, the relationship between the dental personnel and the patient is important in building trust and having a positive psychological impact on the patient's state of mind. Periodontal initial treatment was investigated as to whether the patient's psychological state was altered using the State-Trait Anxiety Inventory psychological test. The results showed that periodontal initial treatment improved the state anxiety levels [2].

Oral health is related to psychosocial, economic, and other factors [3]. Furthermore, oral health differed by age, gender, and other factors [4]. However, these reports do not examine the true feelings and background of the patients. For this reason, qualitative studies were considered useful. Furthermore, as quantitative studies cannot be analyzed, a qualitative study was used for this study.

Qualitative research requires coding words of interest in the data, words outside the data to paraphrase, words to explain theme and concept, and storylines and theoretical descriptions are described. Step for Coding and Theorization (SCAT) is fitted for explicit procedures. SCAT is a qualitative analysis system that integrates individual information. This method has recently been applied in the dentistry field [5]. Data analysis for this study was carried out using SCAT [5–7].

The purpose of this study was to analyze the first-visit behavior of a patient in the maintenance phase of periodontal disease, and the qualitative analysis was performed using SCAT.

## 2. Case Presentation

This case study was approved by the Kyushu Dental University Ethics Committee (No. 16-37). The interviewer obtained informed consent from the patient, including consent for the study and to publish the findings. The patient was given an ID, and the information obtained in this study was recorded on an ID card to ensure subject anonymity.

### 2.1. Diagnosis and Etiology

A 50-year-old female patient was diagnosed with periodontitis (Stage II Grade B) at the Kyushu Dental University Hospital. She had no systemic disease and no history of taking drugs.

### 2.2. Treatment Objectives

Her chief complaint was bleeding of the gingiva at the mandibular teeth. She required periodontal treatment.

### 2.3. Treatment Alternatives

The interview was conducted on a one-to-one basis between the patient and the interviewer. The interview was carried out using a semistructured interview technique in a dental unit at the Kyushu Dental University Hospital. The interview focused on the patient's reasons for visiting the Kyushu Dental University Hospital, reasons for choosing the University Hospital instead of a dental clinic, and reasons for continuing with supportive periodontal therapy (SPT). The interviewer was another dentist, and the interviews were audio-recorded at the dental unit, and the voice recording was transcribed from the tape (SONY Corp., IC Recorder, Tokyo, Japan). The interview time was under 20 min and did not affect the patient's dental treatment.

Texts from the interviews were analyzed qualitatively by SCAT (http://www.educa.nagoya-u.ac.jp/%7Eotani/scat/scatform1.xls, Nagoya, Japan). The SCAT analysis [5] was as follows: (1) extracting words and phrases of attention in the text and data; (2) transforming the extracted words and phrases into extratextual words and phrases; (3) the converted words and phrases are described in the extratextual concept; (4) themes and component concepts are described in the overall context; and (5) describe the storyline and theoretical description from the theme and the component concepts. Thus, the storyline and theory description were obtained from the subject's information.

Periodontal clinical parameters such as probing pocket depth (PPD) and bleeding on probing as a percentage of the measured area (%BOP(+)) were examined at the first visit, and it had been 25 years since her initial visit. PPD measurements of the mesial, central, and distal of the buccal and lingual sides of each tooth were achieved using William's probe (Hu-Friedy·Japan Co., LLC, Tokyo, Japan). The %BOP(+) was considered positive when bleeding occurred within 30 s after probing.

### 2.4. Treatment Progress

Periodontal treatment consisted of oral hygiene instruction, scaling, root planing, and periodontal surgery. It had been 25 years since the initial visit, and she had undergone maintenance for 21 years.

### 2.5. Treatment Results

Theoretical descriptions obtained from the SCAT analysis included not healed after several visits to the dental clinic, complaints about the dental clinic, the good relationship between the dental personnel and patient, and the importance of SPT (Table 1).

Table 2 shows the comparison of periodontal tissue from the initial visit and 25 years since the initial visit. Periodontal treatment achieved significant improvement in periodontal tissue, with the average PPD decreasing from 3.3 mm at the initial visit to 2.7 mm after 25 years and the average %BOP(+) decreasing from 34.7% at the initial visit to 13.2% after 25 years (Table 2).

## 3. Discussion

Periodontal treatment consists of oral hygiene instruction, scaling, root planing, and periodontal surgery. Plaque control is very important in periodontal treatment; however, depending on the patient's character and concerns, it is often difficult to maintain patient motivation over long periods of time. Various factors affect the patient's particular physical and cultural environment [8]. The success of periodontal treatment is influenced by differences in patient personality tendencies and motivation; therefore, standard methods do not always yield good results. Qualitative studies are suitable for analyses of such patient backgrounds [9]. If periodontal treatment is to be successful, it may be necessary to know the personality and psychopathology of the patient. Because periodontal treatment requires long-term treatment and patient motivation, it is necessary to build trust through a positive relationship between the dental personnel and the dentist at every visit, thus increasing the patient's motivation for dental treatment.

Axelsson, Nyström, and Lindhe [10] reported that if the periodontal maintenance had begun, periodontal tissue did not return, but if the periodontal maintenance had not started, periodontal tissue returned. However, it is often difficult for patients to remain motivated for maintenance. In this particular study, the patient's honest opinions, personality, and background could not be determined as it was a qualitative research study. Therefore, we selected a qualitative study to investigate these factors.

Qualitative research uses observation and interviews to collect descriptive data and analyze language and concepts. Types of qualitative research include the grounded theory approach, content analysis, and SCAT. This study used SCAT, which was adapted to explicit procedures and small data. SCAT increases the abstraction of text, creates a thematic composition, and concludes storylines and theories.

From the present study, an initial negative theoretical description was obtained. The patient had been to several dental clinics previously, but her periodontitis had not healed, and she had complained about the quality of dental care. Negative feelings about dental treatment may have resulted from a combination of various factors formed in her past experiences. On the other hand, a positive theoretical description was also achieved the patient became more comfortable with dental treatment. The subsequent relationship between the dental personnel and the patient was good, and the patient understood the importance of periodontal maintenance therapy.

Toothbrushes have the greatest impact on oral hygiene in periodontal maintenance. Oral health education acknowledges the influence of wider factors alongside interpersonal and individual influences which should be taken into consideration. Therefore, oral hygiene education is important [11, 12].

In the future, periodontal maintenance including toothbrush instruction should incorporate the dental professional–patient relationship and the psychosocial aspects of oral health [11, 12].

In addition to conventional periodontal treatment, minimally invasive approaches such as probiotics, paraprobiotics, ozone, and postbiotics can help maintain tissue integrity and prevent disease progression [13, 14]. Incorporating these proactive measures into the patient's maintenance regimen can help ensure long-term periodontal stability and improve overall oral health.

This study had one subject. There may have been another cause. Therefore, we plan to increase the number of studies and continue to research and scrutinize our findings in the future.

## 4. Conclusion

A good relationship between the dental personnel and the patient led to successful long-term maintenance therapy. Appropriate periodontal treatment, a good relationship with the dental personnel, and the incorporation of minimally invasive approaches appear to be important factors in increasing patient maintenance consultations and ensuring long-term periodontal stability.

## Figures and Tables

**Table 1:** Qualitative data analysis using SCAT (Steps for Coding and Theorization).

**Number**	**Speaker**	**1. Extracting words and phrases of attention in the text and data**	**2. Transforming the extracted words and phrases into extratextual words and phrases**	**3. The converted words and phrases are described in the extratextual concept**	**4. Themes and component concepts are described in the overall context**	**5. Describe the storyline and theoretical description from the theme and the component concepts**
1	Interviewer	Hello. It has been 25 years since you last visited this hospital.	25 years passed.	25 years in hospital.	Long-term dental consultations.	
2	Subject	In that time, I have been to several dental clinics and my gingiva has not healed.	Consulted several dental clinics.	Left mandibular molar does not heal.	Complain about dental clinic.	Deal with dental treatment and identify the dental clinic.
30	Interviewer	Continue maintenance in the future. Periodontal maintenance is an arduous treatment, so let us do our best.	The importance of routine maintenance	The importance of SPT		
31	Subject	I will do my best.	Let us do our best together.	I will continue the SPT.	I will continue treatment in the future.	I will continue treatment in the future.
Storyline (what you can say at the moment)		The patient came to the clinic spontaneously; periodontal disease was diagnosed and healed. Increased motivation for periodontal treatment. Good relationship between the dental personnel and the patient. The patient wishes to continue to attend the Kyushu Dental University Hospital.				
Theoretical description		No healing after visits to several dental clinics, complaints about the dental clinics, good relationship between the dental personnel and the patient, and the importance of SPT				
Research points Issues		Does the subject understand periodontal disease? Does the subject understand the relationship between periodontal disease and the whole body?				

*Note:* Download the form from the SCAT website: http://www.educa.nagoya-u.ac.jp/%7Eotani/scat/scatform1.xls.

**Table 2:** Comparison of periodontal tissue at the initial visit and 25 years since the initial visit.

	**Initial**	**25 years since the initial visit**
Ave PPD (mm)	2.3	2.7
%BOP(+)	34.7	13.2

Abbreviations: %BOP(+), bleeding on probing as a percentage of the measured area; Ave PPD, probing pocket depth.

## Data Availability

The data used to support the findings of this study are included within the article.

## References

[1] Junge T., Topoll H., Eickholz P., Petsos H. (2021). Retrospective long‐term analysis of tooth loss over 20 years in a specialist practice setting: periodontally healthy/gingivitis and compromised patients. *Journal of Clinical Periodontology*.

[2] Muraoka K., Kubota K., Amano M., Yokota M. (2005). Change in STAI during initial treatment of periodontitis. *Journal of Psychosomatic Oral Medicine*.

[3] Watt R. G. (2007). From victim blaming to upstream action: tackling the social determinants of oral health inequalities. *Community Dentistry and Oral Epidemiology*.

[4] Sabbah W., Tsakos G., Chandola T., Sheiham A., Watt R. G. (2007). Social gradients in oral and general health. *Journal of Dental Research*.

[5] Kato T., Sugiyama S., Makino M., Naito T. (2006). Qualitative research on the background of long-term maintenance patients. *The Japanese Journal of Conservative Dentistry*.

[6] Goto A., Rudd R. E., Lai A. Y. (2014). Leveraging public health nurses for disaster risk communication in Fukushima City: a qualitative analysis of nurse’s written records of parenting counseling and peer discussions. *BMC Health Services Research*.

[7] Hirayama Y., Otani T., Matsushima M. (2017). Japanese citizen’s attitude toward end-of-life care and advance directives: a qualitative study for members of medical cooperatives. *Journal of General and Family Medicine*.

[8] Patrick D. L., Lee R. S. Y., Nucci M., Grembowski D., Jolles C. Z., Milgrom P. (2006). Reducing oral health disparities: a focus on social and cultural determinants. *BMC Oral Health*.

[9] Kanno K., Kawai Y., Kobayashi K. (2006). Behavioral study of denture acceptance part1 qualitative investigation of linguistic data and validity of coding process. *Nihon Hotetsu Shika Gakkai Zasshi*.

[10] Axelsson P., Nyström B., Lindhe J. (2004). The long-term effect of a plaque control program on tooth mortality, caries and periodontal disease in adults. *Journal of Clinical Periodontology*.

[11] Barnes E., Bullock A., Chestnutt I. G. (2022). What influences the provision and reception of oral health education? A narrative review of the literature. *Community Dentistry and Oral Epidemiology*.

[12] Preda C., Butera A., Pelle S. (2021). The efficacy of powered oscillating heads vs. powered sonic action heads toothbrushes to maintain periodontal and peri-implant health: a narrative review. *International Journal of Environmental Research and Public Health*.

[13] Butera A., Gallo S., Pascadopoli M., Luraghi G., Scribante A. (2021). Ozonized water administration in peri-implant mucositis Sites: a randomized clinical trial. *Applied Sciences*.

[14] Butera A., Gallo S., Pascadopoli M., Taccardi D., Scribante A. (2022). Home oral care of periodontal patients using antimicrobial gel with postbiotics, lactoferrin, and aloe barbadensis leaf juice powder vs. conventional chlorhexidine gel: a split-mouth randomized clinical trial. *Antibiotics*.

